# In Situ Growth of CoS Nanosheets on Carbon Fiber Surfaces to Enhance the Interfacial Properties of Carbon Fiber/Norbornene Polyimide Composites

**DOI:** 10.3390/ma18102334

**Published:** 2025-05-17

**Authors:** Guoqiang Kong, Jianshun Feng, Fengjie Qi, Meng Shao, Qiubing Yu, Guang Yu, Xin Ren, Wenjie Yuan, Qifen Wang, Wenbo Liu, Xiang Zhao, Dayong Li, Xuejun Hou, Bo Zhu

**Affiliations:** 1 Shandong Institute of Nonmetallic Materials, Jinan 250031, China; 2 Key Laboratory for Liquid Solid Structural Evolution and Processing of Materials (Ministry of Education), School of Materials Science and Engineering, Shandong University, Jinan 250061, China

**Keywords:** carbon fiber, PI-NA, CoS nanosheet, mechanical lock-in effect, interfacial strength

## Abstract

This study presents a novel method for altering the surface properties of carbon fiber (CF) to improve the bonding strength at its interface with norbornene–polyimide (PI-NA) resin. Cobaltous sulfide (CoS) nanosheets were successfully synthesized on the CF surface using a solvothermal method combined with a chemical sulfidation process. The modification increased the specific surface area and surface roughness of the CFs, enhancing the interfacial mechanical lock-in effect between the fibers and the resin. This facilitated effective load transfer between the resin and the fibers, thereby significantly improving the interfacial strength of CF-reinforced polymers (CFRPs). The experimental findings showed that after solvothermal treatment with a precursor solution of 0.006 g/mL for 4.5 h, vertical CoS nanosheets were successfully grown on the CF surface. The interlaminar shear strength (ILSS) and interfacial shear strength (IFSS) of the modified CF reached 60.03 MPa and 83.27 MPa, respectively, representing increases of 19.49% and 27.01% compared to untreated fiber composites. This research demonstrates that this method is simple to apply and promising in terms of industrial scalability.

## 1. Introduction

Carbon-reinforced polymer composite material (CFRP) is mainly composed of carbon fiber (CF) as a reinforcement and resin matrix as a binder. Due to its low coefficient of thermal expansion, high strength, high specific strength, high specific modulus, and excellent corrosion resistance, it is widely applied in the aerospace and automotive industries and the low-altitude economy [1,2,3,4]. However, during the preparation of CF, high-temperature carbonization leads to the formation of highly crystalline graphite planes, resulting in a lower number of surface-active functional groups and a strong chemical inertness [5,6,7]. As a result, the adsorption and wettability between the CF and the polymer matrix are poor, leading to a weak interfacial bond. This weak interfacial adhesion makes CF composites prone to delamination and fracture under external stress [8,9,10]. Therefore, enhancing the interfacial strength of CFRPs is key to improving their overall performance.

The interfacial bonding between fibers and resins is typically categorized into physical bonding, which is dominated by mechanisms such as covalent bonding, weak molecular forces, hydrogen bridge bonds, and mechanical bonding [11,12,13,14]. Therefore, the focus of interfacial research is on improving the wettability between the fiber and resin matrix, along with enhancing the mechanical interlocking and chemical bonding at the interface to effectively improve the overall performance of the composite material. Based on the two types of bonding—physical and chemical—recent studies have explored various surface modification techniques for treating CF, including chemical grafting, electrochemical oxidation, plasma treatment, and nanomaterial modification [15,16,17,18]. These methods aim to enhance the adhesion strength between CF and the resin matrix, thereby effectively improving the interfacial stability. In the methods mentioned above, chemical grafting refers to the introduction of functionalities onto the fiber surface, which enhances the covalent or non-covalent interactions between the fiber and matrix, thereby improving the bonding strength between the CF and the resin. For example, Sun et al. used caprolactam to block the isocyanate bonds of 3-isocyanopropyltriethoxysilane (IPTS), grafted the modified coupling agent onto surface-oxidized CF under optimized hydrolysis conditions, and fabricated CF-reinforced polyamide 6 composites with a 23.83% increase in interlaminar shear strength [19]. Dabees et al. grafted 4-nitroaniline and 4-aminophenol onto the surface of CF. Through non-covalent interactions between the grafted molecules and the sulfur groups in polyphenylsulfide, the interfacial adhesion between the CF and polyphenylsulfide was enhanced [20]. Xu et al. employed an in situ polymerization method to introduce an amine-capped poly(cyclotriphosphazene-co-4,4′-oxydianiline) coating onto the CF surface, thereby markedly enhancing its wettability and reactivity and ultimately improving the interfacial strength of CF/epoxy resin composites by approximately 70.5% [21]. Wu et al. employed a mussel-inspired co-deposition method to co-deposit ferric ion–polydopamine (PDA) onto the surface of CF. During this process, strong coordination bonds were formed between Fe^3+^ and catechol groups, which enhanced the interfacial crosslinking density, improved stress transfer, and thereby improved the interfacial shear strength (IFSS) of the composite material [22].

The nano-species modification method involves introducing nanoscale particles (such as carbon nanotubes (CNTs), zinc oxide (ZnO), Mxene, manganese dioxide (MnO_2_), and graphene oxide (GO)) to the fiber surface to enhance its surfaceness and specific surface area, thereby promoting mechanical interlocking between the fiber and the polymer matrix, ultimately improving their interfacial bonding strength [23,24,25,26,27]. For example, Cai et al. introduced cerium oxide nanoparticles (CeO_2_NPs) onto the CF surface by hydrolyzing ammonium cerium nitrate, thereby increasing the specific surface area and surface energy of the CF and enhancing the interfacial adhesion strength between the CF and epoxy resin [28]. Fakhrhoseini et al. modified CF by growing Fe_3_O_4_ magnetic nanoparticles (MNPs) at a high temperature of 1000 °C, which enhanced the mechanical interlocking between the CF and the polymer matrix, significantly improving the interfacial shear strength [29]. Li et al. employed a hydrothermal method to in situ grow organic metal framework (MOF) nanosheets on the CF surface using a layer-by-layer approach, thereby increasing the surface roughness and surface energy of the CF and ultimately improving the interfacial properties of the composite materials [30]. Additionally, Li et al. modified the fiber surface by grafting functionalized GO, which increased the surface energy of the CF and effectively improved the interfacial adhesion strength between the CF and bismaleimide (BMI) [31]. Quan et al. successfully synthesized ZnO nanoparticles on the CF surface with sodium alginate serving as the synthesis medium. The introduction of the ZnO nanolayer increased the interfacial area and the surface roughness of the fibers, thereby enhancing the mechanical interlocking and load transfer between the fibers and the resin matrix [27]. These studies demonstrate that introducing nanomaterials onto the surface of CF can effectively enhance the surface energy and specific surface area of the CF, thereby improving the wettability and mechanical bonding between CF and a polymer matrix. Hence, this study presents an approach to modify the composite interphase by in situ growing cobaltous sulfide (CoS) nanosheet vertical arrays via a hydrothermal method. With the construction of these CoS nanosheet arrays, the CF surface becomes rougher, facilitating enhanced mechanical lock-in effects and wettability with PI-NA resin and resulting in markedly improved interfacial adhesion.

## 2. Preparation and Experiment

### 2.1. Preparation

#### 2.1.1. CF Desizing

The CF based on PAN bundles was placed in a high-temperature oven, heated at a rate of 10 °C/min to 380 °C, and maintained at this temperature for 2 h. After naturally cooling to room temperature, the CF bundles were washed three times with deionized water to remove surface carbon. The resulting carbon fibers were designated as De-CF.

#### 2.1.2. Formulating Precursor Solutions Used in CF Surface Treatment

The preparation method for CoS nanosheet precursor solutions was based on previous research [32]. This method involved preparing solutions with different concentrations to obtain three distinct CoS nanosheet precursor solutions. Initially, a base solution, A, was prepared by mixing 100 mL of deionized water with 100 mL of diethylenetriamine (DETA) and stirring for 30 min until homogeneous. Subsequently, using solution A as the base, three precursor solutions of different concentrations were prepared:

1. Solution B (solute mass concentration of 0.002 g/mL): 0.318 g of CoCl_2_·6H_2_O and 0.1 g of thioacetamide (TAA) were added to solution A and stirred at 1000 rpm for 60 min until completely dissolved.
2. Solution C (solute mass concentration of 0.006 g/mL): 0.954 g of CoCl_2_·6H_2_O and 0.3 g of TAA were added to another portion of solution A and stirred under the same conditions for 60 min until completely dissolved.
3. Solution D (solute mass concentration of 0.008 g/mL): 1.272 g of CoCl_2_·6H_2_O and 0.4 g of TAA were added to a third portion of solution A and stirred at 1000 rpm for 60 min until completely dissolved.

Through these steps, three CoS nanosheet precursor solutions (B, C, and D) with different concentrations were successfully prepared, laying the foundation for the subsequent synthesis of CoS nanosheets.

### 2.2. Experiment

#### 2.2.1. Surface Oxidation and In Situ Growth of CoS Nanosheets on CF

Numerous hydroxyl and carboxyl groups (-OH and -COOH) were introduced onto De-CF surfaces via a 60 °C H_2_O_2_-mediated oxidation process maintained for a 2 h duration, and the oxidized CF was named CFO. The oxidation treatment of CF surfaces introduces oxygen-containing functional groups, which serve as effective adsorption sites for the growth of CoS nanosheets. Subsequently, the CFO was dipped into the pre-synthesized precursor solutions (solutions B, C, and D), and each solution was transferred into a 500 mL reaction vessel. The reaction was conducted at 180 °C for 4.5 h. After the reaction was completed, the CFs were washed by alternately soaking them in deionized water and ethanol to remove any residual solvents and products not loaded onto the surface of the CF. The CFs were then dried at 120 °C for 8 h. The treated fibers were named CFO-S1, CFO-S2, and CFO-S3, respectively.

#### 2.2.2. Fabrication of CF/PI Composites

As depicted in Figure S1a, the CF/PI composite was fabricated through a multi-step process. The De-CF, CFO, CFO-S1, CFO-S2, and CFO-S3 substrates (24 bundles each) were first impregnated with a PI-NA resin precursor at a controlled CF-to-resin mass ratio of 6:4, forming a uniform prepreg layer. Post-drying (45 °C/12 h) eliminated residual solvents from the prepreg layer. Following drying, the prepregs were placed into a mold and subjected to heating and compression to form the CF/PI composites. The composite molding procedure followed a precise thermal profile with four sequential temperature stages: 120 °C (held for 100 min), 200 °C (held for 90 min), 280 °C (held for 60 min), and 320 °C (held for 240 min). Pressure application occurred during the 280 °C stage. The resulting composites were designated as De-CF/PI, CFO/PI, CFO-S1/PI, CFO-S2/PI, and CFO-S3/PI.

## 3. Results

**Figure 1:**
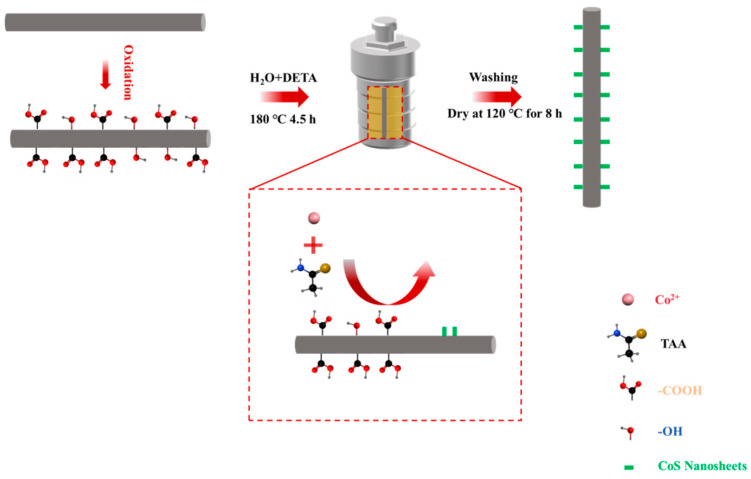
Mechanism of CoS nanosheet growth on CF surface.

As illustrated in Figure 1, treatment of the CF with 30% H_2_O_2_ solution resulted in the formation of numerous oxygen-containing functional groups, including -COOH and -OH, on the CF surface. These functional groups provided essential active sites that facilitated the subsequent in situ growth of CoS nanosheets. Then, Co^2+^ ions were adsorbed onto the CF surface through electrostatic interactions with the -OH and -COOH. These adsorbed cobalt ions then reacted with thioacetamide to form CoS nanosheet structures under water–thermal conditions.

**Figure 2:**
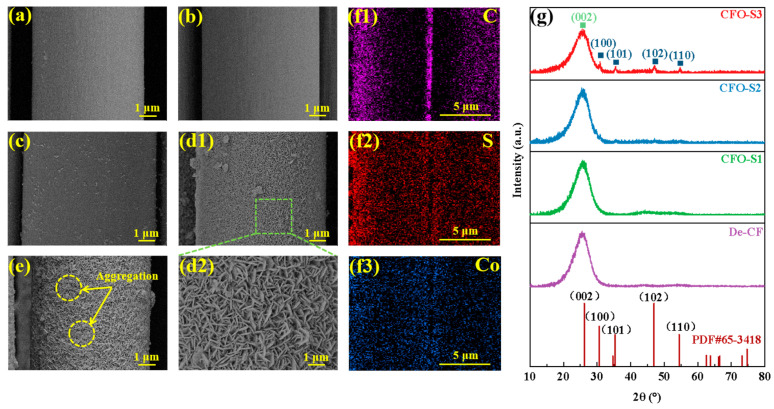
SEM images: (**a**) De-CF; (**b**) CFO; (**c**) CFO-S1; (**d1**,**d2**) CFO-S2; (**e**) CFO-S3; (**f1**–**f3**) EDS elemental mapping of CFO-S2 at low magnification; (**g**) XRD analysis of crystalline phases present in CF samples.

As shown in Figure 2a, residual carbon deposits remained on the CF surface after high-temperature desizing. However, after treatment with hydrogen peroxide solution, as depicted in Figure 2b, these residues were completely removed, leaving a distinct recess on the surface. As shown in Figure 2(d1,d2), when the concentration of the precursor solution was 0.002 g/mL, only a small amount of CoS nanoparticles formed on the surface of the treated CF. With an increase in precursor concentration to 0.006 g/mL (as shown in Figure 2(d1,d2)), the CF surface became uniformly covered with vertically grown CoS nanosheets. When the concentration was further increased to 0.008 g/mL (as shown in Figure 2e), although the CoS nanosheet structures still formed, significant aggregation occurred on the surface, resulting in cluster-like accumulations in certain regions. The results can be attributed to the limited nucleation of CoS at a precursor concentration of 0.002 g/mL during the hydrothermal reaction, where both sulfur and cobalt sources are insufficient for crystal growth. This leads to the formation of only a small amount of disordered CoS nanoparticles on the CF surface. As the precursor concentration increases to 0.006 g/mL, the supply of sulfur and cobalt sources becomes adequate, promoting the formation of a continuous nanosheet structure on the CF surface. However, when the precursor concentration is further increased to 0.008 g/mL, the excessive generation of CoS nanosheets leads to aggregation, resulting in the occurrence of clustered deposition in certain areas. EDS elemental mapping (Figure 2(f1–f3)) confirmed the co-existence of C, S, and Co elements throughout the CFO-S2 sample, validating the successful synthesis of CoS nanostructures on the CF surface. A crystallographic analysis via XRD patterns (Figure 2g) revealed that all samples maintained the characteristic (002) diffraction peak of graphitic carbon at 2θ = 25.3°, indicating preservation of the fundamental CF structure despite surface modifications [33]. The diffraction peaks at 30.6°, 35.3°, 46.9°, and 54.5° correspond to the (100), (101), (102), and (110) planes of hexagonal CoS (PDF#65-3418), respectively. The XRD patterns reveal a concentration-dependent intensification of CoS diffraction peaks. The highest-concentration treatment (CFO-S3) resulted in substantially stronger diffraction signals at the characteristic (100), (101), (102), and (110) crystallographic planes of CoS compared to the lower-concentration treatments (CFO-S1 and CFO-S2) [34]. These results confirm that increasing the treatment concentration promotes higher loading of CoS nanostructures on the CF surface.

**Figure 3:**
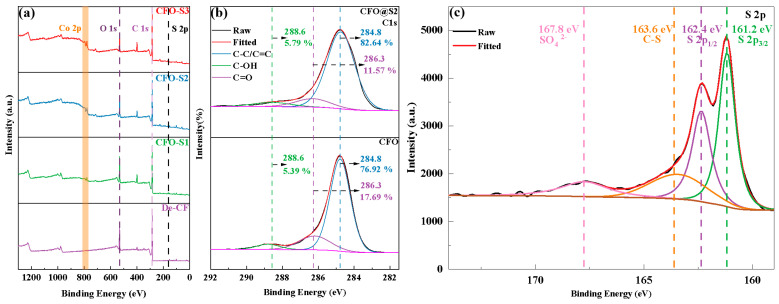
(**a**) Wide-scan XPS spectra; (**b**) C 1s peak spectra of CFO and CFO@S2; (**c**) S 2p peak spectra of CFO-S2.

The XPS spectra displayed in Figure 3a reveal compositional differences among the various fiber specimens under investigation. In the case of CFO-S1, CFO-S2, and CFO-S3 samples, peaks corresponding to S 2p were observed in the 160–170 eV range, and Co 2p peaks were detected between 760 and 820 eV, indicating that CoS nanostructures were successfully loaded onto the CF surface. Additionally, with an increase in the precursor concentration, the intensities of the C, O, and Co peaks increased, indicating that the loading amount of CoS on the CF surface also increased. As shown in Figure 3b, the peaks at 284.8 eV, 286.3 eV, and 288.6 eV correspond to C-C/C=C, C-OH, and C=O, respectively. For the CFO sample, the content of C-OH was 17.69%, and the content of C=O was 5.39%. After hydrothermal treatment and the growth of CoS nanosheets, the C-OH content decreased to 11.57%, while the C=O content remained almost unchanged at 5.79%. This change can be attributed to the hydrothermal treatment, during which the -OH groups on the CF surface are disrupted, leading to a decrease in the C-OH content. The XPS spectrum in Figure 3c exhibits characteristic peaks at 161.2 eV (S 2p_3/2_), 162.4 eV (S 2p_1/2_), 163.6 eV (C-S bond), and 167.8 eV (SO_4_^2−^). Of particular significance is the peak at 163.6 eV representing the C-S bond, which suggests that chemical bonding may exist between the CoS nanosheets and the CF surface [32].

**Figure 4:**
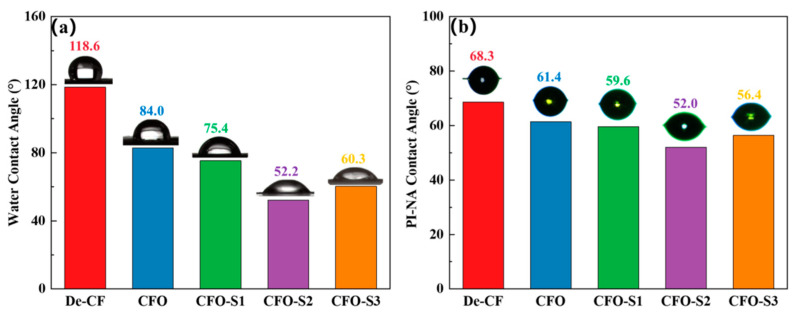
(**a**) Water and (**b**) PI-NA precursor solution contact angles.

The wetting behavior at the CF–resin interface is fundamentally determined by the surface affinity characteristics of the CF. To quantify the alterations in CF surface properties before and after CoS nanosheet modification, all CF specimens underwent a contact angle assessment using both the sessile drop technique and the single-fiber contact angle method. As shown in Figure 4a, the water contact angle of De-CF was 118.6°, whereas the water contact angle of CFO decreased to 84.0°. This result is due to the untreated CF’s smooth surface and the absence of polar groups. During the oxidation treatment, oxygen-containing groups were introduced to the surface, enhancing the surface affinity and thus reducing the contact angle. When CFO was modified with a 0.002 g/mL precursor solution, the water contact angle of CFO-S1 exhibited a progressive decline to 75.4°, which is significantly lower than that of De-CF (118.6°). This can be attributed to the formation of CoS nanoparticles on the surface of CFO-S1, which increases the surface roughness and creates more wettability sites, facilitating easier spreading of the liquid. Upon elevation of the precursor solution concentration to 0.006 g/mL, the measured wetting angle for the CFO-S2 specimen demonstrated an additional reduction, reaching 52.2°. This is due to the formation of CoS nanosheet structures on the fiber surface, allowing the liquid to penetrate the micropores or gaps in the nanosheets through capillary action, thus significantly reducing the contact angle [35,36]. At a precursor solution concentration of 0.008 g/mL, the contact angle of CFO-S3 slightly increased to 60.3°. This phenomenon is attributed to the thicker and more aggregated CoS nanosheets, which affect the spreading behavior of the liquid droplet. To further investigate the impact of CoS nanosheet modification on the wetting behavior of CF in PI-NA solution, fiber contact angle tests were conducted on different modified CF samples. According to Figure 4b, De-CF exhibited a contact angle of 68.3° with PI-NA solution. Meanwhile, CF samples modified using precursor solutions of different concentrations showed contact angles with PI-NA solution of 59.6° (CFO-S1), 52.0° (CFO-S2), and 56.4° (CFO-S3). This trend is consistent with the results of the water contact angle tests. The contact angles of the modified CF samples are significantly lower than that of untreated CF, which is primarily due to the formation of CoS nanostructures on the CF surface. This increases the surface roughness, thereby enhancing the surface energy and promoting the infiltration of the PI-NA solution.

**Figure 5:**
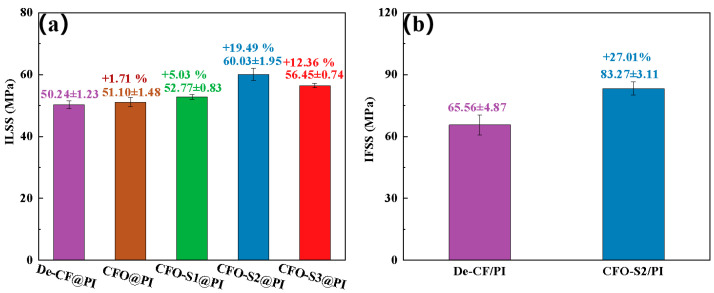
(**a**) The ILSS of the CF/PI composites; (**b**) IFSS of De-CF/PI, CFO-S2/PI.

The mechanical resistance characteristics of CF/PI composites are predominantly determined by the interfacial adhesive efficacy at the fiber–matrix boundary junction. In this study, ILSS testing was utilized to assess and compare the interfacial adhesion between the fiber and PI-NA resin matrix, both before and after surface modification. Figure 5a indicates that unmodified De-CF/PI composites exhibited an ILSS value of 50.24 MPa. The ILSS value of CFO/PI was 51.1 MPa, which is only a slight increase (1.71%) compared to that of De-CF/PI. This modest improvement is likely due to the increased polarity of the CF after oxidation, which facilitates better resin impregnation, thereby enabling the resin to better adhere to the defects on the fiber surface. The experimental steps and data have been updated in the manuscript for your review. Following treatment with a 0.002 g/mL precursor solution, the resulting CFO-S1/PI composites showed a minimal improvement in ILSS, reaching only 52.77 MPa. The minimal improvement in ILSS observed in CFO-S1/PI composites can be explained by the limited formation of CoS nanoparticles on the fiber surface under low-concentration precursor treatment. These sparse nanoparticles failed to create effective mechanical interlocking with the resin matrix. However, increasing the precursor solution concentration to 0.004 g/mL resulted in CFO-S2/PI composites with a substantially enhanced ILSS value of 60.03 MPa—a 19.49% increase over that of De-CF/PI composites. This remarkable improvement stems from the development of vertically oriented CoS nanosheets on the fiber surface, which substantially enhanced mechanical interlocking with the PI resin matrix. These nanosheets effectively impeded crack propagation at the interface during stress-induced failure, consequently enhancing the interlaminar properties of the CFO-S2/PI composite. However, when the precursor solution concentration was further increased to 0.008 g/mL, the ILSS value of the CFO-S3/PI composite reached 56.45 MPa, showing a 12.36% improvement compared to that of De-CF/PI. Such performance improvement stemmed primarily from the physical interlock mechanism occurring at the interface between resin matrix and the CoS nanosheets deposited on the fiber’s exterior surface. Nevertheless, it is significant to observe that CFO-S3/PI showed a 6.34% lower ILSS value than CFO-S2/PI. This performance degradation could be attributed to the formation of excessively thick CoS nanosheet structures with local agglomeration on the CF surface under higher-concentration conditions, which hindered effective contact between the PI-NA resin and CF. Consequently, when the composite was subjected to stress, it failed to effectively transfer the stress to the CF surface, promoting the initiation and propagation of interfacial cracks, ultimately leading to interlaminar delamination and decreased ILSS values. The bonding strength at the boundary between treated/untreated fibers and the resin matrix was quantified using IFSS testing. The results presented in Figure 5b indicate that while the control De-CF/PI composite showed an IFSS of 65.56 MPa, the CFO-S2/PI composite reached a substantially higher value of 83.27 MPa—a significant improvement of 27.01%. This remarkable enhancement in interfacial performance can be attributed to the successful construction of continuous CoS nanosheets on the CF surface. The enhanced surface roughness of the fibers, resulting from these nanostructures, reinforced the mechanical anchoring between fiber and resin interfaces, consequently delivering significant improvements in interfacial adhesion strength [37,38].

**Figure 6:**
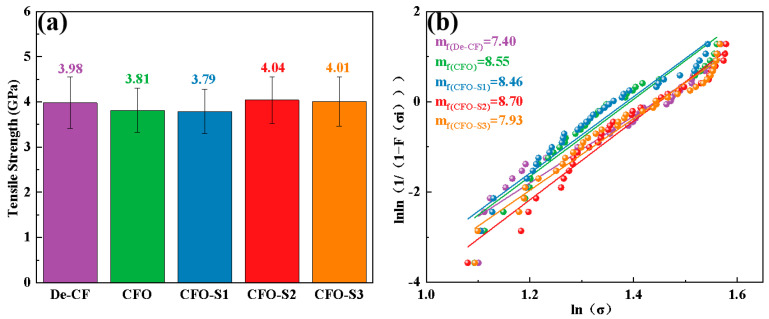
(**a**) Single-fiber tensile strength measurements for all CF samples and (**b**) corresponding Weibull distribution fitting.

The mechanical response of fiber to different surface modification techniques was systematically investigated through tensile strength testing of individual filaments in their pristine and modified states, with the comparative results depicted in Figure 6a. Desized CF exhibited a tensile strength of 3.98 GPa. Following additional oxidation treatment, the tensile strength of a single CFO filament decreased to 3.81 GPa. When the CF was subjected to hydrothermal treatment with a precursor solution concentration of 0.002 g/L, the resulting CFO-S1 sample exhibited a tensile strength of 3.79 GPa. As the precursor solution concentration increased to 0.006 g/L, the tensile strength of the CFO-S2 sample improved to 4.04 GPa. These findings suggest that increasing the precursor solution concentration led to the formation of a vertically grown CoS nanosheet network on the CF surface. The formation of these nanoscale structures effectively repaired surface defects on the CF, reducing stress concentrations under load and consequently enhancing its tensile strength. However, when the precursor solution concentration was further increased to 0.008 g/L, the tensile strength of the CFO-S3 sample slightly decreased to 4.01 GPa. It is believed that this slight decrease may be due to partial aggregation of the CoS nanosheet structure, leading to an uneven load distribution, ultimately causing a minor reduction in tensile strength. The Weibull distribution was applied to assess the variability in the strength of CF monofilaments, as illustrated in Figure 6b. m_f_ values serve as metrics for assessing the consistency and homogeneity of single-fiber strength. The m_f_ value of CFO obtained after oxidation treatment was 8.55, which was higher than the value for De-CF (7.4), indicating that although the oxidation etching effect of hydrogen peroxide reduces the tensile strength of the CFs, it improves their stability. The m_f_ values of CFO-S1, CFO-S2, and CFO-S3 were 8.46, 8.70, and 7.93, respectively. Compared to De-CF, the CFs treated with the precursor solution (CFO-S1, CFO-S2, and CFO-S3) exhibited smaller variability. Specifically, when the concentration of precursor solution was 0.006 g/L, CFO-S2 had a higher m_f_ value than the other concentrations. This is attributed to the continuous three-dimensional CoS nanosheet structure, which effectively strengthens the CF surface, thereby improving the stability of the monofilaments. However, as the concentration of precursor solution increased to 0.008 g/L, the m_f_ value of CFO-S3 decreased, likely due to the aggregation of nanoplates on the fiber surface, leading to an uneven stress distribution and, consequently, a reduction in the stability of the CF.

**Figure 7:**
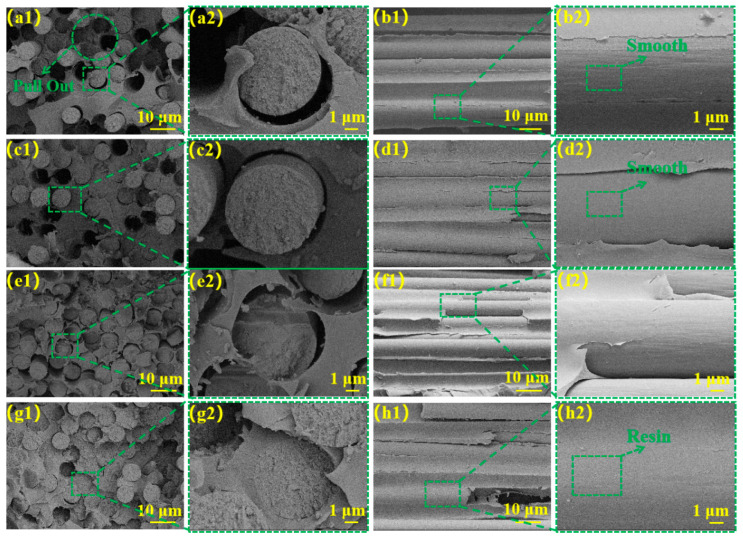
Radial cross-sectional structural SEM images: (**a1**,**a2**) De-CF/PI composites, (**c1**,**c2**) CFO/PI, (**e1**,**e2**) CFO-S1/PI, and (**g1**,**g2**) CFO-S2/PI; latitudinal cross-sectional structure: (**b1**,**b2**) De-CF/PI, (**d1**,**d2**) CFO/PI (**f1**,**f2**) CFO-S1/PI, and (**h1**,**h2**) CFO-S2/PI.

The morphological characteristics of radial and transverse fracture surfaces were investigated using scanning electron microscopy (SEM), providing insights into the damage mechanisms and structural deterioration of CF/PI composites when subjected to loading conditions. Figure 7(a1,a2) illustrates that the radial cross-sectional morphology of De-CF/PI composite revealed abundant hollow regions resulting from fiber detachment and considerable interfacial voids separating fibers from the resin matrix.

The transverse cross-section (Figure 7(b1,b2)) revealed smooth fibrous interfaces containing vacant interstitial spaces between filaments. These observations indicate inadequate bonding at the interface of the fibers and PI-NA. Consequently, when the composite is subjected to stress, the load cannot be effectively transferred from CF to the resin matrix, resulting in stress concentration at the fiber–resin interface. This leads to interfacial delamination and ultimately results in deteriorated ILSS and IFSS properties of the De-CF/PI composite materials. As shown in Figure 7(c1,c2), the surface of the CFO/PI composite exhibited a smooth appearance with minimal residual resin matrix. Furthermore, as shown in Figure 7(d1,d2), the fiber pull-out phenomenon was alleviated compared to that for De-CF/PI. This result can be attributed to the lack of reactive groups on the fiber surface that form chemical bonds with the resin, which means that the interfacial strength is not significantly enhanced. However, the increased polarity of the fibers after oxidation promotes resin impregnation, thereby improving the bond between the resin and the fibers, which slightly alleviates the fiber pull-out phenomenon.

A cross-sectional fracture plane analysis (Figure 7(e1,e2)) indicated that CFO-S1/PI composites demonstrated significantly reduced fiber-detachment-induced voids relative to De-CF/PI composites, although partial fiber–resin interfacial gaps remained present. Illustrated in Figure 7(f1,f2), an observation of the weft cross-section of CFO-S1/PI demonstrated slight residual polymer matrix on the fiber exteriors. Such observational findings suggest that the interfacial mechanical bond between the fiber and PI-NA resin was moderately enhanced, thereby improving interfacial stability under mechanical loading. A transverse fracture surface analysis (Figure 7(g1,g2)) revealed minimal fiber pull-out in CFO-S2/PI composites, indicating strong fiber–resin interfacial bonding. An examination of the longitudinal fracture surface (Figure (7h1,h2)) demonstrated substantial resin adhesion on fiber surfaces with complete resin impregnation in the inter-fiber regions. These observations confirm the formation of an uninterrupted and stable CF–resin interface within CFO-S2/PI composites, significantly hindering crack growth. This interfacial enhancement stems from the mechanical bonding force formed between the CF and matrix, which strengthens the fiber–resin bonding and enables efficient stress transfer from matrix to fiber, consequently improving both the ILSS and IFSS of the composites.

**Figure 8:**
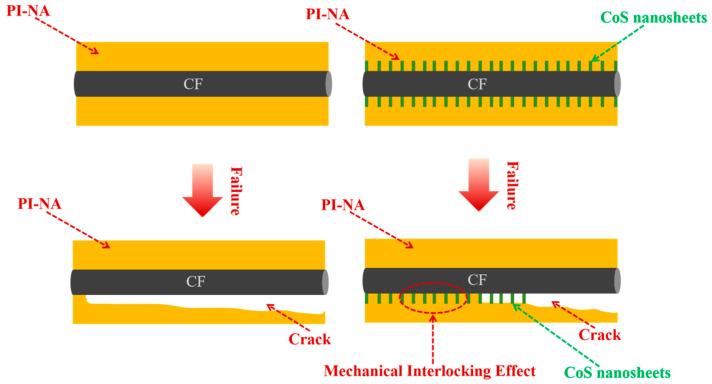
Schematic illustration of failure mechanism of De-CF/PI and CFO-S2/PI.

The interfacial failure mechanism plays a crucial role in determining the overall performance of composite materials at their interfaces. As depicted in Figure 8, when De-CF/PI composites undergo mechanical stress, the insufficient bonding between the CF and resin facilitates crack propagation along the fiber surface. This weak interfacial bonding ultimately leads to premature composite failure under comparatively reduced interlayer shear loading conditions. In the case of CFO-S2/PI composites, the vertically grown CoS nanosheets significantly reinforce structural anchoring at the CF/PI interface, establishing three-dimensional fiber–matrix interconnection. This optimized architecture enables efficient load transmission from the polymer matrix to reinforcing fibers, thereby improving the material’s capacity for load distribution and energy dispersion. Consequently, fracture advancement is effectively restrained, resulting in superior interlaminar shear performance.

## 4. Conclusions

The present investigation explored the formation mechanism governing CoS nanosheets on CF surfaces and proposed a strategy to reinforce interfacial bonding strength at the CF/PI interface using these CoS nanosheets. Spectroscopic analyses (XRD/XPS) demonstrated that CoS nanosheets homogeneously grew on the CF substrates. SEM, BET, and contact angle tests revealed that these CoS nanosheets effectively increased the surface roughness of the CF, thus improving its wettability. Mechanical testing results showed that, compared to those of untreated CF, the ILSS and IFSS of CFO-S2 composites increased by 19.49% and 27.01%, respectively. This study indicates that CoS nanosheets anchored to CF substrates enhance the mechanical interlocking and fiber–matrix interfacial adhesion mechanisms between CF and PI-NA resin by increasing the CF’s surface roughness. This method is simple to apply and promising in terms of its industrial scalability.

## Data Availability

The original contributions presented in this study are included in the article/Supplementary Material. Further inquiries can be directed to the corresponding author.

## Supplementary Materials

The following supporting information can be downloaded at: https://www.mdpi.com/article/10.3390/ma18102334/s1. Figure S1: (a) Preparation schematic of CF/PI composite laminates; (b) ILSS samples and (c) ILSS test schematic diagram; Figure S2: (a) Picture of IFSS samples (b) Process picture of IFSS test; Figure S3: Schematic of carbon fiber monofilament tensile sample preparation; Figure S4: Sample preparation and test method for water contact angle.; Tabe S1: Definition.
