# Supplementary material for: In Situ Growth of CoS Nanosheets on Carbon Fiber Surfaces to Enhance the Interfacial Properties of Carbon Fiber/Norbornene Polyimide Composites
Source: Materials (Basel). 2025 May 17;18(10):2334. doi: 10.3390/ma18102334 (PMC12113463; doi:10.3390/ma18102334)
Supplement: Supplementary file 1 [file materials-18-02334-s001.zip › materials-3565408-supplementary.pdf]

In Situ Growth of CoS Nanosheets on Carbon Fiber Surface to Enhance the Interfacial Properties of Carbon Fiber/norbornene polyimide Composites

Guoqiang Kong[1], Jianshun Feng[2], Fengjie Qi[1], Meng Shao[1], Qiubing Yu[1], Guang Yu[1], Xin Ren[1], Wenjie Yuan[1], Qifen Wang[1], Wenbo Liu[1], Xiang Zhao[1], Dayong Li[1], Xuejun Hou[1], Bo Zhu[2]*

Author Affiliations:

[1] Shandong Institute of Nonmetallic Materials, Jinan, 250031, Shandong, China.

[2] Key Laboratory for Liquid Solid Structural Evolution and Processing of Materials (Ministry of Education), School of Materials Science and Engineering, Shandong University, Jinan 250061, China

*Corresponding authors

E-mail: zhubo@sdu.edu.cn

Materials

Cobalt chloride hexahydrate ($CoCl_2 \cdot 6H_2O$, 99 %), thioacetamide (TAA, 99.9 %) and diethylenetriamine (DETA) were purchased from Macklin Biochemical Co., Ltd., Shanghai, China. CF (T700) was obtained from Guangwei Expansion Fiber Co., Ltd., Weihai, China. Absolute alcohol (AR, 99.5 %) was purchased from Xuran Biotechnology Co., Ltd., Jinan, China. Deionized water was prepared in-house. PI-NA resin precursor solution (50 wt%) was sourced from the Institute of Chemistry Chinese Academy of Sciences, located in Beijing, China. Hydrogen peroxide (H_2O_2, 30 wt%) solution was supplied from Hebei Jiocui Environmental Protection Technology Co., Ltd., located in Handan, China.

Experimental Characterization

ILSS:

The CF/PI laminates were sectioned to form specimens measuring 20 × 10 × 2 mm, as shown in Fig. S1(b). The ILSS of these composites was evaluated using a short beam test conducted with a Universal Testing Machine (CMT4204, MTS-SANS, Donghua University, China), following the JC/T 773-2010 standard. In this three-point bending test, each specimen was placed on supports with a 10 mm span and subjected to a central bending load at a rate of 1 mm/min, as depicted in Fig. S1(c). The test was terminated either upon specimen failure or when the maximum load was reached, at which point the failure mode and peak

load were recorded. Each sample underwent at least five tests, and the ILSS was calculated as the average of these measurements. The formula used to determine ILSS is provided below:

$$\tau_M = 3P/4bh \quad (1)$$

In the above formula: τ_M represents the short beam strength (MPa), P denotes the failure load (N), b corresponds to the specimen width (mm), and h indicates the specimen thickness (mm).

IFSS:

The IFSS of CF/PI was evaluated using a micro-bond test (model HM410, Tokyo, Japan). The specimen fabrication and experimental protocols are illustrated in Fig. S2. In the first step, a CF monofilament was secured onto an iron frame. A 10 wt% PI-NA precursor solution was then prepared and applied to the CF monofilament by brushing, which subsequently led to the formation of droplets on the fiber surface due to surface tension. The iron frame with the coated CF was subsequently placed in an oven, where it underwent a thermal treatment following a step-wise heating protocol of 120 °C, followed by 200 °C, then 280 °C, and finally 320 °C, with one-hour duration at each temperature point. This heating process facilitated the conversion of the PI-NA precursor to PI-NA on the CF surface and ensured the evaporation of the solvent. After heat treatment, the carbon filament was removed from the metal support structure, mounted onto a paper frame, and placed on the

interfacial performance testing device. During the test, a symmetrical droplet with a diameter of 40-60 μm was selected, and the pulling rate was set to 1 μm/s. Each sample underwent a minimum of five tests, and the average IFSS was calculated. The IFSS was quantified by measuring the peak tension force attained during the microdroplet detachment process. The mathematical expression employed for this determination is presented below:

$$IFSS = F/\pi dl \quad (2)$$

where F is the maximum load (N) when the fiber is pulled out, d is the diameter of the fiber filament (m), and l is the length of the resin droplet (m).

Single-Fiber Tensile Testing:

The tensile strength of the fibers was evaluated using a Fiber Tensiometer (XQ-1C, XinXn, China) in accordance with the GB/T 31290-2022 standard for stretching CF monofilaments. The preparation of fiber tensile specimens is depicted in Fig. S3. Each CF monofilament was anchored to a cardboard frame, establishing a testing span of 20 mm. This paper was then mounted onto the testing device, and tensile tests were performed at a rate of 2 mm/min. For each group of samples, a minimum of 35 fibers were tested, and the average tensile strength was calculated. The tensile strength (σ) was determined using the following formula:

$$\sigma = 4F/\pi d^2 \quad (3)$$

where σ represents the tensile strength of a single CF (GPa), F is the maximum breaking load (N), and d is the diameter of the single CF (m). Statistical assessment of the experimental data was conducted through implementation of Weibull probability analysis.

SEM testing

Field emission scanning electron microscopy (FE-SEM, SU-70 model, JEOL, Ibaraki, Japan) was employed to investigate the surface morphology of the CF and the fracture surfaces of the composite materials. A 15 kV voltage was applied during the SEM analysis. Before imaging, the samples were coated with a thin layer of gold for 30 seconds using sputter coating.

XPS Testing

X-ray photoelectron spectroscopy (XPS, AXIS ULTRA, HORIBA Jobin Yvon, Paris, France) was used to examine the changes in elemental composition and functional groups on the surface of the CF. X-ray emission was produced by a non-monochromatic ultra-high vacuum (UHV) source using the Mg Kα line (12 kV, 200 W) as the anode. After an initial survey scan, a high-resolution scan was performed at a pass energy of 10 eV to analyze the chemical state of the surface, including the chemical composition and valence states of the elements. The excitation energy applied was 1253.6 eV. To prevent charging effects during

analysis, the fiber-shaped samples were cut to appropriate sizes and mounted onto the sample holder with copper tape.

Contact Angle

The water contact angle of the fibers was evaluated using the sessile drop method, as depicted in Fig. S4. Initially, the carbon filament bundle was meticulously positioned in a planar configuration and secured adjacently onto microscope slides, applying tension to ensure a smooth and even top surface. The fibers were densely arranged in close proximity without interstitial spaces. The mounted specimens were subsequently oriented in a level position upon the measurement platform of a Contact Angle Meter(JC2000D1, Kezhong, Shanghai, China) for measurement. A water droplet of approximately 10 μL was placed on fiber tow, and once the droplet stabilized for 1 s, a photograph was taken to capture the contact angle. wetting behavior was quantified through computational analysis involving five-point elliptical curve-fitting algorithms applied to the droplet photo. Furthermore, the contact angle of the PI-NA precursor solution on the fiber monofilament was evaluated employing the single-filament contact angle method. The procedure involved diluting a 50% PI-NA precursor solution to 10% with anhydrous ethanol. The fiber monofilament was then fixed onto rigid cardboard, and the diluted precursor solution was uniformly applied to its surface, forming small droplets. The treated monofilament was placed in an oven at 40 °C to

evaporate the solvent, resulting in resin microdroplets adhering to the fiber surface. These resin droplets were subsequently subjected to contact angle measurement using the ellipse fitting method to accurately determine their contact angles.

XRD

The phase composition of the modified fiber samples was analyzed using X-ray diffraction (XRD). The measurements were performed with a Ni filter and a Cu Kα radiation source, operating at an accelerating voltage of 40 kV and a current of 50 mA. Scanning was conducted at a rate of 10° per minute over a range of 10° to 90°.

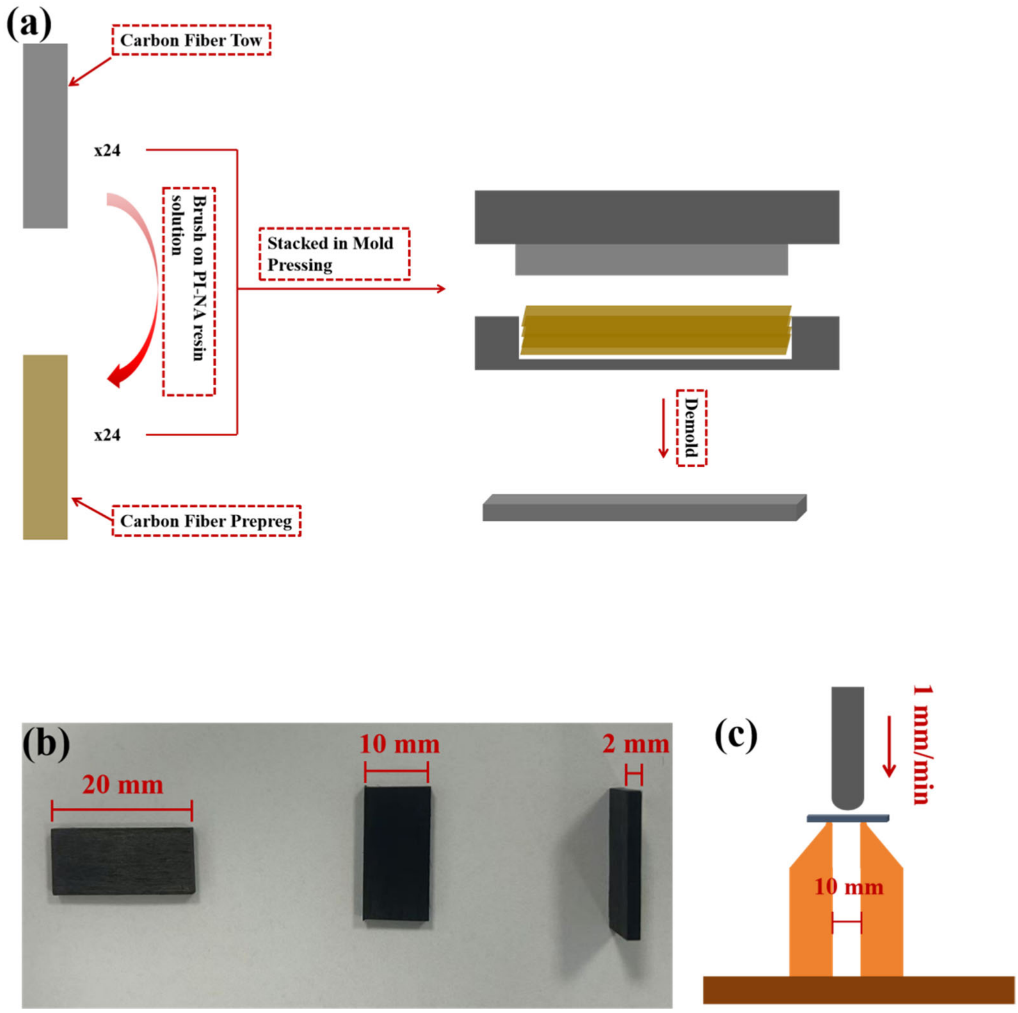

Figure S1: (a) Preparation schematic of CF/PI composite laminates; (b) ILSS samples and (c) ILSS test schematic diagram.

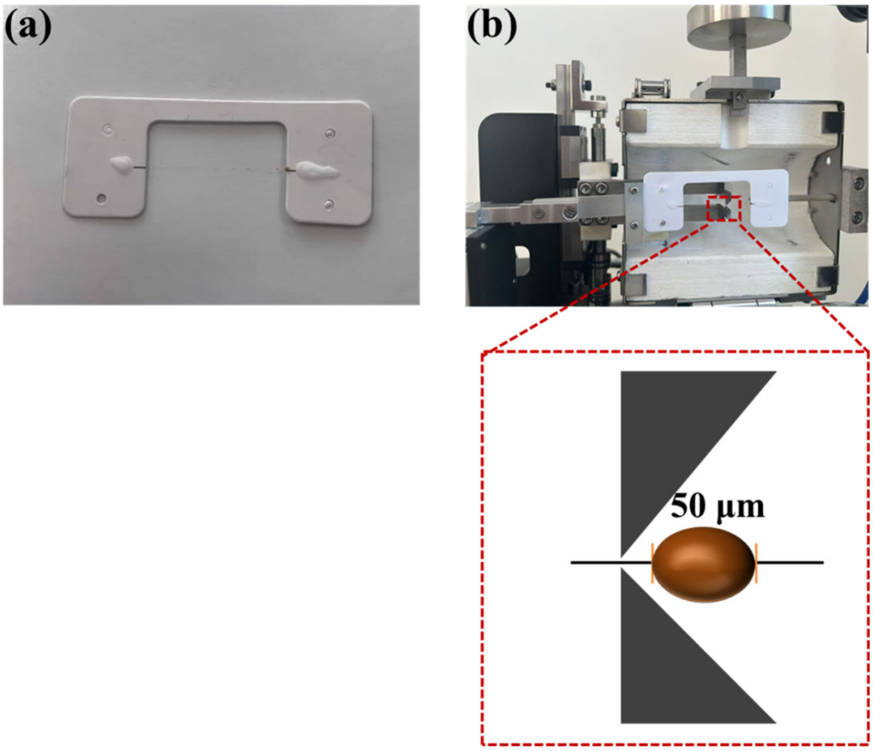

Figure S2: (a) Picture of IFSS samples (b) Process picture of IFSS test.

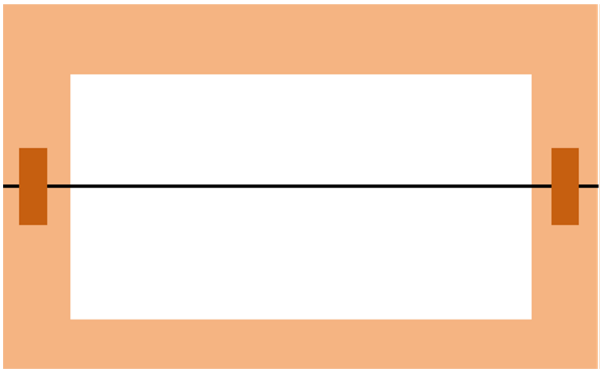

Figure S3: Schematic of carbon fiber monofilament tensile sample preparation.

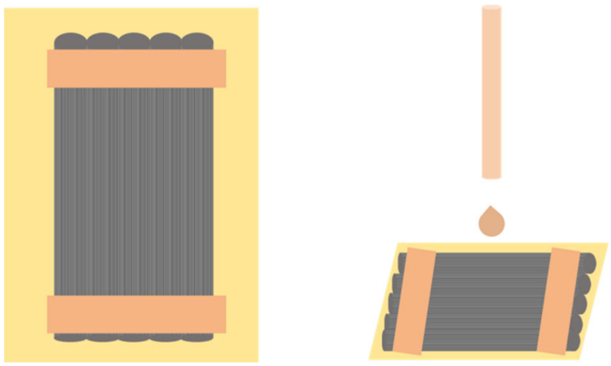

Figure S4: Sample preparation and test method for water contact angle.

Tabe S1：Definition.

Sample name	Name Explanation	Characteristics
De-CF	The sample obtained after desizing treatment of carbon fiber.	After the removal of the sizing agent, the carbon fiber surface appears inert and relatively smooth.
CFO	The sample obtained by further oxidizing the desized carbon fiber using a hydrogen peroxide solution.	After oxidation treatment, the fiber surface is rich in oxygen-containing functional groups.
CFO@S1	The carbon fiber sample obtained by hydrothermal treatment of the CFO sample using a 0.002 g/mL precursor solution of $CoCl_2·6H_2O$ and TAA.	Cobalt sulfide nanoparticles are distributed on the fiber surface.
CFO@S2	The carbon fiber sample obtained by hydrothermal treatment using a 0.006 g/mL precursor solution	Vertically aligned cobalt sulfide nanosheets are

	of $CoCl_2 \cdot 6H_2O$ and TAA.	uniformly grown on the fiber surface without noticeable agglomeration.
CFO@S3	The carbon fiber sample obtained by hydrothermal treatment using a 0.008 g/mL precursor solution of $CoCl_2 \cdot 6H_2O$ and TAA.	Vertically aligned cobalt sulfide nanosheets on the fiber surface exhibit a certain degree of agglomeration.
CFO@S1/PI	The composite material fabricated based on CFO@S1.	/
CFO@S2/PI	The composite material fabricated based on CFO@S2.	/
CFO@S3/PI	The composite material fabricated based on CFO@S3.	/
De-CF/PI	The composite material fabricated based on De-CF.	/
